# Parents’ emotion socialization behaviors in response to preschool-aged children’s justified and unjustified negative emotions

**DOI:** 10.1371/journal.pone.0283689

**Published:** 2023-04-19

**Authors:** Lauren G. Bailes, Garrett Ennis, Sarah M. Lempres, David A. Cole, Kathryn L. Humphreys

**Affiliations:** 1 Department of Psychology and Human Development, Peabody College, Vanderbilt University, Nashville, Tennessee, United States of America; 2 Department of Psychology, University of Texas at Austin, Austin, Texas, United States of America; 3 Department of Psychiatry and Behavioral Sciences, Duke University, Durham, North Carolina, United States of America; Babes-Bolyai University: Universitatea Babes-Bolyai, ROMANIA

## Abstract

Parental socialization of children’s negative emotions is believed to contribute to children’s emotional development, with supportive, process-oriented responses (e.g., explicit acknowledgment of emotional expression and emotion processing) providing opportunities for children to experience and develop adaptive emotion regulation strategies for negative emotions. On the other hand, non-supportive, outcome-oriented responses (e.g., minimizing or punishing children for negative emotional expressions) tend to undermine such opportunities. Less clear, however, is the degree to which parents’ own emotional and cognitive processes influence their emotion socialization behaviors. In particular, the *perceived justifiability* of children’s negative emotions may be an important factor for parents’ socialization behaviors as parents may only attend to emotional displays that they feel are reasonable. Using a sample of 234 mothers and fathers (parents of 146 unique preschool aged children), we examined the degree to which parents reported: (1) feeling specific emotions as a function of whether they viewed children’s negative emotional expressions; (2) engaging in emotion socialization behaviors as a function of whether they viewed children’s negative emotions. Last, we examined whether parents’ reported emotions were related to their behaviors. For caregivers’ emotions and behaviors, we examined whether patterns differed as a function of whether the children’s emotions were perceived as justified or unjustified. Parents were more likely to report feeling emotions such as anger and frustration when they viewed children’s negative emotions as *unjustified* relative to justified, and for these unjustified negative emotions, anger and frustration were related to more outcome-oriented behaviors. Emotions such as sadness and guilt, however, were related to more process-oriented behaviors, regardless of whether parents felt children’s negative emotions were justified or unjustified. Findings highlight the interrelatedness of emotional and cognitive processes within the parenting context and their potential influence on emotion socialization behaviors.

## Introduction

Current conceptualizations of emotion socialization primarily delineate between two types of parental behaviors in response to children’s negative emotions: supportive emotion socialization (e.g., encouragement of emotion and emotion processing) and non-supportive emotion socialization (e.g., emotion minimizing and punitive responses) [1, 2]. Within this conceptualization, supportive emotion socialization behaviors are postulated to provide children with opportunities to learn how to identify, express, and regulate their emotions, whereas non-supportive emotion socialization behaviors, in contrast, may hinder the development of adaptive emotion regulation. Over time, supportive parental responses to children’s negative emotions are linked to less intense negative affect [3–5], greater emotional self-awareness [3, 5], and more adaptive physiological regulation [6, 7]. Non-supportive emotion socialization behaviors have been longitudinally associated with increased risk for internalizing behaviors [8, 9] and externalizing behaviors [9]. Although grouping emotion socialization parenting behaviors as supportive and non-supportive is potentially useful clinically, this broad framework is limited in that it does not consider whether specific parenting behaviors contribute to emotion regulation strategies that children learn to employ. Recently, emotion socialization researchers have proposed shifting from the categorization of behaviors as understood as supportive or non-supportive to a framework that considers how the behavior impacts children’s emotional development [10]. One such attempt at expanding our understanding of emotion socialization behaviors has come from attachment theorists/interventionists who describe “Being With” children’s emotions as being attuned to, validating, and comforting the child as they experience negative emotions [11, 12]. These “Being With”, or *process-oriented* responses reflect the parent being physically close to the child and supporting their exploration of emotions (e.g., holding the child and saying, “Can you tell me why you are so sad right now?”). Alternatively, *outcome-oriented* responses, characterized by parenting behaviors aimed at ending the negative emotional experience without aiding in the regulation of children’s emotions (e.g., distracting or walking away from the child). These responses may undermine opportunities for children to learn adaptive emotion regulation skills [6]. Whereas supportive and non-supportive emotion socialization categories are based on whether the caregiving behaviors alleviate distress, a process- vs. outcome-oriented approach to categorizing emotion socialization better delineates behaviors theorized to affect the development of adaptive emotion regulation.

Emotions influence both caregiving behaviors and related cognitions. Dix’s Affective Organization of Parenting theory [13] highlights the importance of parents’ emotions and their ability to regulate them within the parenting context as a reflection of the quality of the caregiving environment. The emotion arousal and regulation processes that caregivers experience during parent–child interactions are postulated to affect their availability and responsiveness to their children’s emotions [13, 14]. A great deal of research has centered on broad classes of emotions (i.e., positive and negative valence) and parenting. Mothers’ negative emotions during caregiving interactions have been associated with less sensitive and responsive caregiving [15–17]. However, other work, including that by Dix and colleagues [15], have examined the role of mothers’ discrete emotions during a free play and clean-up task with 1 year old children, and found that maternal worry, sadness, anger, and guilt were differentially associated with maternal behaviors. While maternal worry for the child was associated with more synchronous behavior and less restrictive control (e.g., attempts to get children to conform to mothers’ demands and wishes); maternal sadness, anger, and guilt were associated with more asynchronous behavior. Further, anger has been related to more restrictive control [15], lower sensitivity [18, 19], higher harsh parenting [20], and over-reactive discipline [21]. These findings suggest that parents’ specific emotions impact caregiving behaviors, and that understanding the cognitive processes that underlie parents’ emotions may be fruitful for understanding variation in caregiving behaviors.

Social information processing theories posit that there is an intricate and reciprocal link between emotions and cognitions. Parents’ emotions within parent–child interactions are, in part, driven by their beliefs about and interpretations of their children’s behavior [22]. For example, a crying child may elicit sympathetic feelings and a supportive parental response if the caregiver believes the child to be justifiably upset (e.g., clear cause or source of the negative emotion such as hunger or an injury). However, if the child’s negative emotion appears to be unjustified (e.g., all physical needs have been met, environment is suitable), the caregiver may feel frustrated or irritated, responding in a way to discourage their child’s display of negative emotion (e.g., distraction, walking away; [19, 20]). Studies have shown that parental cognitions about their children can have direct and indirect effects on the parent–child relationship and child development [23–25]. Negative parenting cognitions have been found to attenuate more harsh and severe punishment [26–29], influencing parental affective responses and socialization strategies, [27, 30] related to maladaptive child behaviors, including aggression [31]. Additionally, there is evidence that caregivers’ attributions and children’s behaviors are transactional in nature given that mothers’ hostile attributions about their children’s behavior was associated with more child negative behavior, which was in turn, associated with more hostile attributions [24, 25]. Taken together, it is evident that we can learn much about parenting behavior by better understanding how and what type of cognitions tend to influence caregiving behavior during instances of child distress.

Parenting cognitions and emotions in the context of children’s distress, compared to non-distress, is important to focus on for two reasons. First, children who exhibit distress may be more difficult to care for given that children higher in negative emotionality are reported to be more demanding of their caregivers [32]. Second, sounds of crying and distress often elicit feelings of anxiety and discomfort and physiological reactions [33] and undermine important executive functions such as working memory and inhibitory control [34]. Although crying may make caregiving more challenging, the quality of the caregiving interaction during times of distress may be the most important for children’s developmental outcomes [18]. Drawing on work differentiating between parental sensitivity to distress and non-distress, the evidence is overwhelming that sensitivity to distress is more important for children’s emotion-related outcomes, including emotion regulation and attachment security compared to sensitivity to non-distress [35, 36]. Thus, socialization of negative emotions, compared to positive emotions, may be more important than socialization of positive or neutral emotions for informing children’s emotion regulation and emerging psychopathology [37].

In summary, according to Dix’s [13] theory, caregivers’ organization of cognitions in response to children’s behaviors is important for emotional arousal and regulation, both of which ultimately inform caregivers’ emotion socialization behaviors. In addition to theoretical support, empirical evidence suggests that links between cognitions and emotions are important for caregiving behaviors [32, 34]. To date, however, it is not clear whether parents’ emotions and socialization behaviors are consistent when parents view children’s emotions as justified and unjustified.

The current study had three aims: (1) characterize how likely parents were to feel certain emotions when children’s negative emotions were viewed as justified and unjustified; (2) characterize parents’ emotion socialization behaviors in response to children’s justified and unjustified negative emotions, and (3) examine the association between caregivers’ reported emotions and behaviors in response to children’s (3a) justified and (3b) unjustified negative emotions. This is the first study to assess parents’ emotions and behaviors in response to their children’s *justified* and *unjustified* negative emotions, providing insight to the role of both caregivers’ cognitions and their emotions in response to varied emotional scenarios. Though we hypothesized that justified and unjustified emotions may elicit different emotions and behaviors in parents, these analyses were exploratory and descriptive. In terms of our expectations regarding the associations between parent emotions and behaviors, we expected that parents’ sadness and guilt would be associated with more process-oriented caregiving behaviors (e.g., holding the child, acknowledging the child’s emotion). Parents’ emotions such as anger and frustration would be associated with more outcome-oriented parenting behaviors (e.g., telling the child to stop, walking away from the child), particularly in response to children’s unjustified emotions.

## Method

### Participants

Participants were recruited from two online registries housed at Vanderbilt University consisting of Nashville-area families with interest in participating in research projects within the university and referrals from previous participants. Eligibility requirements included English fluency and having a child between 3.00 and 5.99 years of age. Participants with more than one child in this age range were asked to answer the survey in reference to their oldest child in that range. The first parent to complete the survey was asked to invite a co-parent to also complete the survey about the target child, resulting in a total of 254 parents (136 mothers, 118 fathers; parent age *M* = 35.62 years, *SD* = 4.14 years) of 146 target children (child age *M* = 4.45 years, *SD* = 0.83 years). We included three attention check questions throughout the study. Data from participants who did not answer these questions accurately were removed from the analysis, leaving a final sample of 234 parents (136 mothers, 98 fathers) with from 146 children. Demographic information for the final sample can be found in Table 1.

**Table 1:** Participant demographics.

Variable		Mean (SD) or Mean (Percentage)
**Parent Race**		
	White	219 (94%)
	Black/African American	6 (3%)
	Asian/Asian American	5 (2%)
	Biracial or Multiracial	4 (2%)
**Parent Ethnicity**		
	Hispanic or Latinx	9 (4%)
	Not Hispanic or Latinx	225 (96%)
**Parent Education**		
	High School Diploma/GED	4 (2%)
	Some College	17 (7%)
	Associate’s Degree	9 (4%)
	Trade/Technical School	6 (3%)
	Bachelor’s Degree	90 (39%)
	Graduate Degree	108 (46%)
**Parent Marital Status**		
	Married/Domestic Partnership	226 (97%)
	Single, never married	4 (2%)
	Divorced	4 (2%)
**Parent Employment Status**		
	Employed full time	155 (66%)
	Employed part time	19 (8%)
	Homemaker	33 (14%)
	Military	1 (0.4%)
	Out of work, not looking for work	5 (2%)
	Self-employed	17 (7%)
	Student	3 (1%)
	Other	1 (0.4%)
**Gross Annual Household Income**		
	$5,001–15,000	1 (0.4%)
	$15,001–30,000	4 (2%)
	$30,001–60,000	19 (8%)
	$60,001–90,000	41 (18%)
	$90,001–150,000	93 (40%)
	$150,000–250,000	60 (26%)
	More than $250,000	14 (6%)
	Not reported	2 (1%)
**Number of Children**		
	Only 1 child	45 (19%)
	More than 1 child	189 (81%)

### Procedure & measures

Recruitment, procedure, and measures were all in accordance with and approved by the Institutional Review Board at Vanderbilt University (#201722). Recruitment, procedure, and measures were all in accordance with and approved by the Institutional Review Board at Vanderbilt University (#201722). After verifying eligibility, participants completed an online informed consent form and a battery of questionnaires via REDCap (Research Electronic Data Capture) hosted at Vanderbilt University [38, 39]. Participants completed a battery of questionnaires, including the Comfort, Attunement, and Validation of Emotions as part of a larger study. Participants were compensated with a $20 Amazon gift card for their participation. Participants had the option to include the name of their child’s other caregiver (e.g., partner, ex-partner), and if both caregivers of the child participated, the pair received an additional $10 Amazon gift card.

#### Comfort, Attunement, and Validation of Emotions (CAVE) Measure

Using concepts from the *Circle of Security* [11], we developed the CAVE measure as an assessment of caregivers’ self-reported emotions and behaviors in response to children’s caregiver-directed negative emotions (https://osf.io/58aev/). First, caregivers were asked if their children had ever exhibited three different negative emotions (i.e., anger, fear, sadness) that the caregiver thought were justified. If caregivers indicated that the target child had experienced that emotion, they were asked to report on how likely they were to experience feeling twelve different discrete emotions as a response to their child’s negative emotion (e.g., feel uncomfortable, feel guilty). In addition, caregivers were also asked to report on how likely they were to engage in six different behaviors in response to their child’s negative emotion (e.g., hold their child, tell their child to stop). This procedure of asking for a caregiver’s reported emotional and behavioral responses was repeated for all three emotions when justified, as well as for all three emotions when unjustified. In total, caregivers could respond to the emotions and behavior battery up to 6 times regarding their child’s negative emotions: justified anger, unjustified anger, justified fear, unjustified fear, justified sadness, unjustified sadness. The scale consists of a 6-point Likert rating with values recoded so that values ranged from -3 (*very unlikely*) to 3 (*very likely*), with unsure recoded as 0.

Given the number of variables generated from this questionnaire (i.e., 12 emotions and 6 behaviors in response to three justified and three unjustified children’s negative emotions), we took several steps for data reduction. First, frequencies of whether parents said their children experienced justified and unjustified emotions were examined for anger, fear, and sadness. Over 50% of caregivers said that their children exhibited justified sadness, unjustified sadness, justified anger, and unjustified anger. Given the lower frequencies of justified fear and unjustified fear (40% and 31%, respectively), they were removed from subsequent analyses. Additionally, caregiver-endorsed emotion frequencies were examined. Caregiver reports of feeling attentive, excited, happy, calm, indifferent, and proud were not endorsed frequently, and were removed from subsequent analyses, with parents’ report of feeling guilty, sad, angry, frustrated, stressed, and uncomfortable remaining. Caregivers’ reports of mock/tease one’s child (88% said “very unlikely”) was removed given low frequency of endorsement, resulting in five caregiving behaviors: try to distract my child, tell them to stop feeling the emotion, walk away from them, acknowledge the emotion, and hold my child.

To reduce the data and aid in meaningful interpretation, we conducted a series of exploratory factor analyses to determine the best factor structure for the remaining 20 items related to caregiving behaviors. Final results for the factor analysis are in S1 Table in S1 File. Based on examining the scree plot, a three-factor solution using varimax rotation emerged as the best fitting solution, explaining a total of 56% of the variance. Factor 1 included *acknowledge emotion* and *hold child* for both justified and unjustified anger and sadness (8 items total), both behaviors theorized to help children *process* their negative emotions (henceforth called *process-oriented behaviors*). Factor 2 included *tell child to stop* and *walk away from child* for both justified and unjustified anger and sadness (8 items total); these behaviors are theorized to result in ceasing negative emotional expression (henceforth called *outcome-oriented behaviors*). Lastly, *distraction* items loaded into its own factor (4 items total).

### Data analysis plan

Data used in the subsequent analyses are publicly available (https://osf.io/tnspm/). Analyses were conducted in R version 4.0.2 [40]. Aim 1 (characterize caregivers’ emotions in response to justified and unjustified children’s negative emotion) was addressed by testing a series of dependent-samples t-tests to compare mean differences for emotions that parents reported feeling (i.e., guilt, sadness, stress, frustration, anger, and discomfort) in response to their children’s justified and unjustified negative emotions. Aim 2 (characterize caregivers’ behaviors in response to children’s justified and unjustified negative emotions) was assessed by testing a series of dependent-samples t-tests to compare mean differences between process-oriented, outcome-oriented, and distraction behaviors that parents reported engaging in when their children displayed justified vs. unjustified emotions. Importantly, only parents who said that their child has felt both justified and unjustified negative emotions were included in analyses given that not all parents responded to both justified and unjustified items (e.g., a parent could report that their child does not exhibit unjustified anger, and therefore, not all parents would not have any responses to caregiver self-reported emotions and behaviors in response to children’s unjustified anger). Additionally, for Aims 1 and 2, to address dependency within the data (i.e., potential for two caregivers to report on the same child), follow-up analyses that included only one parent for each child were conducted. In such cases when two caregivers reported on the same child, one caregiver was selected at random for these analyses.

Aim 3 (relation between parenting emotions and parenting behaviors for children’s justified and unjustified negative emotions) was tested using a series of regression models in which categories of emotions (i.e., guilt and sadness; anger and frustration; stress and discomfort) were included as independent variables to test how much variance in parents’ process-oriented, outcome-oriented, and distract behaviors was accounted for by parents’ emotions. Data were nested (i.e., up to two parents reporting about the same child) and were handled using cluster robust standard errors to estimate statistical significance [41] and using the *sandwich* package [42] in *R* Studio. Three sets of models were specified for children’s justified and unjustified emotions; parents’ likelihood of feeling: (1) guilt and sadness, (2) frustration and anger, and (3) stress and discomfort. All models contained child age in months as covariate. Dependent variables of parent behaviors (i.e., process-oriented, outcome-oriented, and distracting behaviors) were entered in separate models and analyses were run for children’s justified and unjustified negative emotions. Standardized and unstandardized parameter estimates adjusted for child age are presented in the tables.

## Results

### Preliminary analyses

Correlations between variables of interest are provided in Tables 2 and 3. Caregiving behaviors between justified and unjustified emotions were moderately to highly correlated for process-oriented, outcome-oriented, and distract behaviors. For justified emotions, process- and outcome-oriented caregiving behaviors were negatively correlated. Distraction was correlated with outcome-oriented, but not process-oriented caregiving behaviors. Similar patterns were observed for children’s unjustified negative emotions. For both justified and unjustified negative emotions, caregivers tended to report feeling multiple emotions, evidenced by positive correlations among most of the emotions.

**Table 2:** Correlations among variables of interest for children’s perceived justified negative emotions.

	1.	2.	3.	4.	5.	6.	7.	8.	9.
1. Process-oriented	1								
2. Outcome-oriented	–.12*	1							
3. Distraction	.05	.41**	1						
4. Guilt	.24**	.17*	.12	1					
5. Sadness	.31**	.15*	.10	.82**	1				
6. Stress	.04	.23**	.29**	55**	.61**	1			
7. Frustration	–.04	.33**	.32**	.40**	.37**	.68**	1		
8. Anger	–.09	.40**	.21**	.29**	.28**	.50**	.70**	1	
9. Discomfort	–.06	.28**	.26**	.42**	.40**	.55**	.57**	.51**	1
10. Child age	–.08	–.01	–.10	.01	.03	.06	–.01	.05	.05

Note.

*p < .05,

**p < .01.

**Table 3:** Correlations among variables of interest for children’s perceived unjustified negative emotions.

	1.	2.	3.	4.	5.	6.	7.	8.	9.
1. Process-oriented	1								
2. Outcome-oriented	–.32**	1							
3. Distraction	.01	.45**	1						
4. Guilt	.21**	–.01	.13	1					
5. Sadness	.31**	–.07	.09	.69**	1				
6. Stress	–.02	.21*	.24**	.38**	.43**	1			
7. Frustration	–.21**	.40**	.29**	.21**	.25**	.72**	1		
8. Anger	–.20**	.45**	.23**	.19**	.15**	.54**	.70**	1	
9. Discomfort	.02	.15*	.26**	.38**	.34**	.58**	.50**	.48**	1
10. Child age	–.17*	.11	–.04	–.02	–.01	.06	.21**	.19**	.03

Note.

*p < .05,

**p < .01.

### Primary analyses

#### Aim 1. What emotions do caregivers report when they interpret their children’s negative emotions as justified vs. unjustified?

Descriptive statistics and paired t-test results of the reported caregiver emotions in response to children’s justified and versus unjustified negative emotions are presented in Table 4. Parents reported that they were more likely to feel emotions such as guilt, sadness, and discomfort in response to their children’s justified emotions compared to unjustified emotions. Additionally, caregivers reported feeling more frustration and anger in response to children’s unjustified negative emotion compared to their justified negative emotions. Differences in caregiver feelings of stress were not significant.

**Table 4:** T-tests for children’s justified and unjustified negative emotions in relation to caregivers’ emotions.

		N	Mean	SD	*t*-test (df)	Cohen’s *d*	95% CI Cohen’s *d*
Guilt	Justified	136	1.13	1.60	15.86 (135)**	1.36	1.13, 1.58
	Unjustified	136	–1.40	1.56			
Sadness	Justified	136	1.13	1.54	11.71 (135)	1.00	0.80, 1.21
	Unjustified	136	–0.72	1.70			
Stress	Justified	136	0.30	1.67	1.78 (135)**	0.12	0.05, 0.29
	Unjustified	136	0.13	1.73			
Frustration	Justified	136	–0.47	1.63	–8.46 (135)**	0.73	0.54, 0.91
	Unjustified	136	0.75	1.48			
Anger	Justified	136	–1.73	1.22	–7.84 (135)**	0.67	0.49, 0.86
	Unjustified	136	–0.68	1.73			
Discomfort	Justified	136	–0.87	1.87	3.91 (135)**	0.34	0.16, 0.51
	Unjustified	136	–1.31	1.82			

*Note*. n = 136 derived from selecting one caregiver randomly when two caregivers were available to address dependency within families. *T*-test is comparing the emotion for justified and unjustified emotions.

*p < .05,

**p < .01.

#### Aim 2. What behaviors do caregivers report when they interpret their children’s negative emotions as justified vs. unjustified?

Descriptive statistics and results of paired t-tests of reported caregiving behaviors in response to children’s justified and versus unjustified negative emotions are presented in Table 5. Parents reported that they were more likely to engage in process-oriented behaviors when emotions were viewed as justified compared to unjustified. Conversely, parents reported that they were more likely to engage in outcome-oriented and distraction behaviors when children’s emotions were viewed as unjustified compared to justified.

**Table 5:** T-tests for children’s justified and unjustified negative emotions in relation to caregivers’ behaviors.

		N	Mean	SD	*t-*test (df)	Cohen’s *d*	95% CI Cohen’s *d*
Process-Oriented	Justified	136	2.42	0.65	10.46 (135)**	0.90	0.70, 1.10
	Unjustified	136	1.47	1.27			
Outcome-Oriented	Justified	136	–1.54	1.64	–9.98 (135)**	0.86	0.66, 1.05
	Unjustified	136	–0.24	1.91			
Distraction	Justified	136	0.11	1.93	–2.19 (135)*	0.19	0.2, 0.36
	Unjustified	136	0.39	1.92			

*Note*. n = 136 derived from selecting one caregiver randomly when two caregivers were available to address dependency within families.

*p < .05,

**p < .01.

#### Aim 3. What is the association between caregivers’ reported emotions and caregiving behaviors in response to children’s justified vs. unjustified negative emotions?

The correlations between parents’ emotions and behaviors are presented in Table 2 for children’s justified emotions and Table 3 for unjustified emotions. *R*^*2*^ values for the regression models are presented in Tables 6 and 7 for the associations between reported caregiving emotions and behaviors for justified and unjustified children’s negative emotions, respectively.

**Table 6:** Proportion of variance accounted for parenting behaviors by emotion groups—children’s justified emotions.

		Process-Oriented				Outcome-Oriented				Distraction			
		β	*p*	*R* ^*2*^	95% CI *R*^*2*^	Β	*p*	*R* ^*2*^	95% CI *R*^*2*^	β	*p*	*R* ^*2*^	95% CI *R*^*2*^
Model 1	Guilt	–0.06	.64	.11	.03, .18	0.15	.16	.03	–.01, .07	0.11	.34	.02	–.01, .06
	Sadness	0.36	.004			0.03	.79			0.01	.20		
Model 2	Anger	–0.12	.14	.02	–.01, .07	0.33	.002	.16	.08, .25	–0.02	.77	.11	.04, .19
	Frustration	0.04	.60			0.10	.29			0.34	< .001		
Model 3	Stress	0.11	.09	.02	–.02, .06	0.12	.15	.09	.02, .16	0.21	.02	.10	.03, .18
	Discomfort	–0.11	.14			0.21	.01			0.14	.12		

*Note*. Although βs are provided, the *R*^*2*^ is the primary metric of interest for these analyses.

**Table 7:** Proportion of variance accounted for parenting behaviors by emotion groups—children’s unjustified emotions.

		Process-Oriented				Outcome-Oriented				Distraction			
		β	*p*	*R* ^*2*^	95% CI *R*^*2*^	Β	*p*	*R* ^*2*^	95% CI *R*^*2*^	β	*p*	*R* ^*2*^	95% CI *R*^*2*^
Model 1	Guilt	–0.02	.75	.12	.04, .21	0.09	.41	.02	–.02, .06	0.12	.24	.01	–.02, .05
	Sadness	0.33	< .001			–0.13	.21			0.01	.91		
Model 2	Anger	–0.08	.48	.06	.001, .13	0.32	.001	.21	.12, .31	0.05	.53	.09	.02, .17
	Frustration	–0.13	.23			0.18	.07			0.27	.007		
Model 3	Stress	–0.17	.03	.01	–.02, .05	0.18	.04	.05	–.004, .11	0.13	.12	.08	.01, .15
	Discomfort	–0.05	.53			0.04	.60			0.18	.04		

*Note*. Although βs are provided, the *R*^*2*^ is the primary metric of interest for these analyses.

#### Perceived justified emotions

Results regarding the directionality of the association between parents’ emotions and behaviors for children’s emotions perceived as justified can be found in Table 2 and the proportion of variance explained can be found in Table 6. Parents’ reports of guilt and sadness for their children’s justified negative emotions were related to more process-oriented behaviors (*r*s = .24 and .31, respectively; *R*^*2*^ = .11, 95% CI [0.03, 0.18]). Guilt and sadness did not account for a significant proportion of the variance for outcome-oriented (*R*^*2*^ = .03, 95% CI [-0.01, 0.07]) or distraction (*R*^*2*^ = .02, 95% CI [-0.01, 0.06]). Parents’ anger and frustration were positively correlated with outcome-oriented parenting behaviors (*r*s = .40 and .33, respectively; *R*^*2*^ = .16, 95% CI [0.08, 0.25]) and distraction (*r*s = .21 and .32, respectively; *R*^*2*^ = .11, 95% CI [0.04, 0.19]), but not process-oriented behaviors (*R*^*2*^ = .03, 95% CI [-0.02, 0.05]). Parents’ reports of stress and discomfort were related to more outcome-oriented (*r*s = .23 and .28, respectively; *R*^*2*^ = .09, 95% CI [0.02, 0.16]) and distraction (*r*s = .29 and .26, respectively; *R*^*2*^ = .10, 95% CI [0.03, 0.18]).

#### Perceived unjustified emotions

Results regarding the directionality of the association between parents’ emotions and behaviors for children’s emotions perceived as justified can be found in Table 3 and the proportion of variance explained can be found in Table 7. Parents’ report of guilt and sadness were positively correlated with process-oriented behaviors (*r*s = .21 and .31, respectively; *R*^*2*^ = .12, 95% CI [0.04, 0.21]). Anger and frustration were negatively correlated with process-oriented behaviors (*r*s = -.20 and -.21, respectively; *R*^*2*^ = .06, 95% CI [0.001, 0.13]) and positively correlated with outcome-oriented (*r*s = .45 and .40, respectively; *R*^*2*^ = .21, 95% CI [0.12, 0.31]) and distraction (*r*s = .23 and .29, respectively; *R*^*2*^ = .09, 95% CI [0.02, 0.17]). Stress and discomfort were positively correlated with outcome-oriented (*r*s = .21 and .15, respectively; *R*^*2*^ = .05, 95% CI [-0.004, 0.11]) and distraction (*r*s = .24 and .26 respectively; *R*^*2*^ = .08, 95% CI [0.01, 0.15]).

## Discussion

Parents’ socialization of their children’s emotions is critical for children’s emotional expression and regulation. Thus, understanding what may influence parents’ responses to their children’s negative emotions is important for children’s emotional development. The current study examined if parents’ emotional and behavioral responses to their preschool-aged children’s negative emotions varied depending on whether parents viewed children’s negative emotions as justified or unjustified. We found that parents reported feeling negative emotions, such as frustration and anger, more strongly when children’s negative emotions were viewed as unjustified compared to justified. Further, we examined the associations between parents’ reported emotions and behaviors in response to children’s justified and unjustified negative emotions. The association between parents’ emotions and emotion socialization behaviors differed when parents viewed the emotions as justified compared to unjustified, particularly when children were reported to be experiencing feelings of guilt, sadness, frustration, and anger. Findings from the current study provide support for Dix’s model in two important ways. First, parental emotions may be more important for understanding variations in the caregiving environment when parents are responding to their children’s negative emotions. For example, parents’ feelings of guilt and sadness in response to children’s negative emotions were only related to process-oriented behaviors, but not outcome-oriented or distraction behaviors. Second, the association between parents’ emotions and emotion socialization behavior differed based on parents’ cognitive processes surrounding children’s negative emotions, providing more support for the interplay between cognitions and emotions within the parenting context. For example, parents who reported feeling more anger and frustration in response to their children’s negative emotions also reported engaging in fewer process-oriented emotions, but only for *unjustified* negative emotions.

Importantly, our work bridges emotion socialization with attachment theory and provides an alternative to examining parental emotion socialization behaviors, such that this approach is centered around how parents promote or undermine opportunities theorized to be important for the development of adaptive emotion regulation. *Process-oriented* caregiving behaviors, including acknowledging the emotion and holding the child, are two behaviors that are thought to support emotional development and emotion regulation by providing the child with an opportunity to “organize their feelings” [11] and experience validation in emotional expression. *Outcome-oriented* behaviors, such as telling the child to stop feeling the emotion or walking away from the child, on the other hand, are not likely to provide opportunities for organization and may encourage emotion regulation strategies such as emotion suppression, which has been associated with the development of psychopathology [43]. Parents who employ outcome-oriented parenting behaviors may have a goal to stop the negative emotional experience, and children who frequently experience these types of caregiving behaviors may rely on self-oriented regulation strategies, such as emotion suppression [44]. Self-oriented emotion regulation strategies are not inherently maladaptive, but previous work has suggested children have more adaptive emotional outcomes when they have a diverse set of emotion regulation strategies in which they can use depending on the context and the availability of a caregiver [45].

Although in our study, we had expected two classes of parenting behaviors to emerge: process-oriented and outcome-oriented, we found that items describing parents use of distraction fit better when considered as a separate factor. The conceptualization of emotion socialization posited by Eisenberg considered distraction as a supportive response to children’s negative emotions. Distraction from this perspective is characterized as a behavior that helps children feel better when they are upset [1, 46]. Thus, we considered that parents’ use of distraction would load with process-oriented behaviors. However, distracting a child by offering a new toy after one breaks may mitigate the distress in the moment; such actions may prevent emotion processing. From that perspective, distraction as a socialization technique may limit the opportunity for emotion identification and labeling, which is a central component of emotion understanding and later adaptive emotional development [47]. Distraction may also communicate to the child that the caregiver is not emotionally available to their distress and that they should redirect their attention away from the distress and onto another object rather than toward their caregiver for help [12]. Overreliance on distraction as a response to children’s negative emotions may undermine the child’s opportunities for practicing important skills needed for adaptive emotional development.

### Do parents’ reported emotions and behaviors differ for children’s justified and unjustified emotions?

Consistent with previous research that has linked parents’ emotions and cognitive processes [22, 48–50], we found that when parents viewed emotions as unjustified, compared to justified, parents were more likely to report more frustration and anger and less guilt and sadness. Further, we found that parents engaged in more process-oriented emotion socialization behaviors (i.e., acknowledging their child’s emotion, holding the child) and fewer outcome-oriented emotion socialization behaviors (i.e., walking away, telling child to stop) when children’s negative emotions were viewed as justified compared to unjustified. Parents’ attributions about whether their children’s negative emotions are justified or unjustified was related to whether they engaged in process- or outcome-oriented behaviors.

### How are parents’ emotions related to emotion socialization behaviors?

Findings from our study also highlight the importance of examining the way parents’ discrete emotions uniquely affect parental emotion socialization behaviors, stepping beyond broader categories of emotion valence groups (e.g., positive or negative emotions). Findings for Aim 3 (the association between parents’ reported emotions and emotion socialization behaviors for children’s justified and unjustified negative emotions) were somewhat consistent with hypotheses and prior literature [28]. Specifically, we found that parents’ different emotions were differentially related to categories of parenting behaviors. Of note, parents’ anger and frustration seemed to be related to all caregiving behaviors (i.e., less process-oriented, more outcome-oriented and distraction), compared to parents’ report of guilt and sadness, which only seemed to be related to process-oriented caregiving behaviors. Importantly, the effect of parents’ emotions on emotion socialization behaviors differed whether parents viewed children’s negative emotions as justified or unjustified. The biggest differences emerged in the relation between parent emotion and process-oriented caregiving behaviors. Specifically, anger and frustration were associated with *fewer* process-oriented caregiving strategies, but only when parents viewed their children’s emotions as unjustified, suggesting that the contexts in which children are displaying negative emotions is relevant for their caregivers’ emotions and behaviors.

Taken together, findings from the current study highlight that parents’ responses to their children’s negative emotions may be coaching their children by only reacting and supporting emotions they view as justified or reasonable in that specific context. By avoiding reinforcing what are deemed as “inappropriate” emotional displays, parents may use different emotion socialization behaviors to shape their children’s behavior regarding the expression of certain emotions based on context (e.g., crying following an injury vs. being asked to split the last cookie with their sibling). Parents who feel more frustration and anger in response to their children’s perceived unjustified negative emotions are less likely to support their children’s processing of their negative emotions. Parents may view these unjustified negative emotions as their children overreacting or that the child is using the emotion as manipulation, and, as a result, may be less inclined to help process and work-through that emotion with their child [51]. Importantly, we can only speculate on the consequences of emotion socialization behaviors and whether “child effects” may be influencing parent reporting. For example, children higher in attention-seeking behaviors may elicit reduced process-oriented emotion socialization from caregivers with a goal of reducing perceived unreasonable displays of emotions [52].

This study contributes to the literature in three important ways. First, we examined the role of parents’ discrete emotions in response to children’s negative emotions. Previous work that has collapsed across *positive* and *negative* emotion valence are missing the nuances of how discrete negative emotions function in parent–child interactions. We found that certain discrete negative emotions functioned alongside others in patterns: guilt and sadness tended to have similar effects on parental behavior; frustration and anger also tended to be similarly associated with parents’ emotion socialization behaviors. Guilt and sadness seemed to promote parental emotion socialization behaviors that support children’s processing of their negative emotions (e.g., holding child and acknowledging the emotion) for both justified and unjustified emotions, whereas more anger and frustration promoted more emotion socialization behaviors which undermined opportunities for emotion processing and understanding, in particular for unjustified negative emotions. Second, we examined the unique role of *distraction* as an emotion socialization behavior, which has previously been grouped with other supportive emotion socialization behaviors [2]. However, interestingly, in our sample, *distraction* not only loaded in its own factor, but the patterns of effects were also more similar to outcome-oriented emotion socialization behaviors than process-oriented behaviors. Although more research is needed to examine the degree to which distraction functions as more process- or outcome-oriented in the degree to which these different behaviors are related to children’s emotional development. Third, our findings provide additional support for the linkage between cognitions and emotions within the parenting context, suggesting that parenting cognitions and emotions are intrinsically linked and should be considered jointly.

Despite the strengths of this study and promising direction for future research, there are limitations that need to be considered. First, all data were collected via self-report and from a single measure. Second, these data were cross-sectional and although the directionality of effects was theoretically supported, we ultimately cannot determine whether parents’ emotions came before their socialization behaviors. Experimental research may better allow for determining temporal precedence related to parental emotions and behaviors, but also in how parental emotions and cognitions are related to each other. Third, there was no way to assess whether parents’ judgements of justifiability were universal or consistent with one another (e.g., the same behavior judged as unjustified by one parent may be judged as justified to another in ways that are nonrandom to the other constructs assessed). Future work using experimental methods, such as hypothetical vignettes with varying degrees in which experts would determine a child’s emotional expression to be justified would allow for control over this aspect of context.

Given the importance of parents’ emotion socialization in relation to children’s adaptive emotional development, a critical need exists to understand what factors may contribute to the caregiving behaviors that parents engage in with their children. Findings from our study highlight the importance of considering how parents’ emotions and cognitive processes are related to the caregiving environment. Further, findings suggest a role for interventions that target aiding caregivers on their own emotions in response to their child’s negative emotion displays. However, many questions remain about how process-oriented, outcome-oriented, and distraction behaviors are empirically associated with children’s emotional development. These findings, coupled with future research, have implications for long-term development regarding adaptive emotion regulation strategies that children learn.

## Supporting information

S1 File Final results from factor analysis of caregivers’ socialization behaviors. (DOCX)
