# Supplementary material for: Parents’ emotion socialization behaviors in response to preschool-aged children’s justified and unjustified negative emotions
Source: PLoS One. 2023 Apr 19;18(4):e0283689. doi: 10.1371/journal.pone.0283689 (PMC10115305; doi:10.1371/journal.pone.0283689)
Supplement: S1 File — (DOCX) [file pone.0283689.s001.docx]

Supplemental Table 1. Final results from factor analysis of caregivers’ socialization behaviors.

| **Item** | | |  |  |  |
| --- | --- | --- | --- | --- | --- |
| **Child Emotion** | **Parenting Behavior** | **Perceived Justifiability** | **Factor 1** | **Factor 2** | **Factor 3** |
| Sadness | Hold | Justified | .48 |  |  |
| Sadness | Acknowledge | Justified | .72 |  |  |
| Sadness | Hold | Unjustified | .53 |  |  |
| Sadness | Acknowledge | Unjustified | .81 |  |  |
| Anger | Hold | Justified | .54 |  |  |
| Anger | Acknowledge | Justified | .75 |  |  |
| Anger | Hold | Unjustified | .55 |  |  |
| Anger | Acknowledge | Unjustified | .83 |  |  |
| Sadness | Tell Stop | Justified |  | .55 |  |
| Sadness | Walk Away | Justified |  | .22 |  |
| Sadness | Tell Stop | Unjustified |  | .77 |  |
| Sadness | Walk Away | Unjustified |  | .47 |  |
| Anger | Tell Stop | Justified |  | .71 |  |
| Anger | Walk Away | Justified |  | .40 |  |
| Anger | Tell Stop | Unjustified |  | .88 |  |
| Anger | Walk Away | Unjustified |  | .52 |  |
| Sadness | Distract | Justified |  |  | .68 |
| Sadness | Distract | Unjustified |  |  | .83 |
| Anger | Distract | Justified |  |  | .81 |
| Anger | Distract | Unjustified |  |  | .74 |

Note: Hold = “hold my child”; Acknowledge = “acknowledge my child’s emotion”; Tell Stop = “tell my child to stop”; Walk Away = “walk away from my child”.
